# Novel Desmin Mutation Causing Myofibrillar Myopathy in a Hmong Family

**DOI:** 10.3389/fneur.2019.01375

**Published:** 2020-01-10

**Authors:** Stefan Nicolau, Benjamin M. Howe, Elie Naddaf

**Affiliations:** ^1^Department of Neurology, Mayo Clinic, Rochester, MN, United States; ^2^Department of Radiology, Mayo Clinic, Rochester, MN, United States

**Keywords:** desmin, myofibrillar myopathy, distal myopathy, hereditary myopathy, muscle MRI

## Abstract

Myofibrillar myopathies (MFM) are a clinically and genetically heterogenous group of inherited myopathies characterized by aggregation of Z-disc proteins. Mutations in desmin account for ~7% of MFM. We report here a Hmong family with an autosomal dominant MFM caused by a novel variant in the desmin gene. The proband presented with lower limb followed by upper limb weakness starting in the 5th decade. On examination, there was distal more than proximal muscle weakness. One sibling was similarly affected, while another had an asymptomatic elevation of creatine kinase. Genetic testing revealed a novel p.Ser13Tyr variant, which was predicted by *in silico* algorithms to alter protein function. Muscle biopsy revealed a MFM. Muscle MRI demonstrated selective involvement of the tensor fasciae latae, semitendinosus, sartorius, gracilis, gastrocnemius, soleus, and peroneus longus muscles. In this family, the histological and MRI findings assisted in the interpretation of genetic testing results.

## Introduction

Myofibrillar myopathies (MFM) are a clinically and genetically heterogenous group of inherited myopathies histologically characterized by aggregation of Z-disc proteins (1). Mutations in 11 different genes have so far been found to cause MFM (2, 3), but approximately half of patients remain without a molecular diagnosis (4). The increasing availability of genetic testing by next-generation sequencing has also led to the identification of many variants of unknown significance (VUS) in genes known to cause MFM. In light of the paucity of functional assays available for most forms of MFM, it is often challenging to determine the pathogenicity of these variants. Muscle MRI however has shown promise in distinguishing certain forms of MFM from other distal myopathies based on patterns of muscle involvement and may similarly be of assistance in the interpretation of VUS (5). We report here a family with an adult-onset distal myopathy caused by a novel heterozygous missense variant in the desmin gene and highlight the role of muscle MRI in interpreting genetic testing results in this family.

## Case Report

The proband was a 47 year-old man of Hmong descent who presented with a 5-year history of progressive lower limb followed by upper limb weakness. Neurological examination revealed severe distal weakness in the upper and lower limbs, along with a mild to moderate degree of proximal weakness. The patient's 50 year-old sister was more severely affected, requiring assistance walking. She started noticing the weakness at age 37, and had no cardiac involvement. A 52 year-old brother had asymptomatic elevated CK level (493 U/L, normal <171). An 80 year-old paternal aunt has become wheelchair bound from the myopathy, which started around age 50. The proband's father had died at age 53 of lung cancer without displaying any signs of neuromuscular disease. Family members were not available for detailed clinical evaluation.

The proband had a creatine kinase level of 669 U/L (normal < 308 U/L). An electromyogram revealed rapid recruitment of complex short duration motor unit potentials in all muscles examined, mixed with long-duration motor unit potentials in some proximal muscles. Fibrillation potentials were present in most muscles. A biopsy of the left quadriceps showed non-specific changes. An echocardiogram did not reveal evidence of a cardiomyopathy, while an electrocardiogram showed a first-degree atrioventricular block.

A next-generation gene panel targeting 121 known myopathy genes revealed a heterozygous VUS in *DES* (c.38C>A; p.Ser13Tyr) and a homozygous VUS in *RYR1* (c. 12880A>G; p.Thr4294Ala). Neither variant was present in population databases, nor known to be disease-causing. The *DES* variant was predicted by three *in-silico* algorithms to adversely affect protein function, while the *RYR1* variant was predicted to be benign.

**Figure 1:**
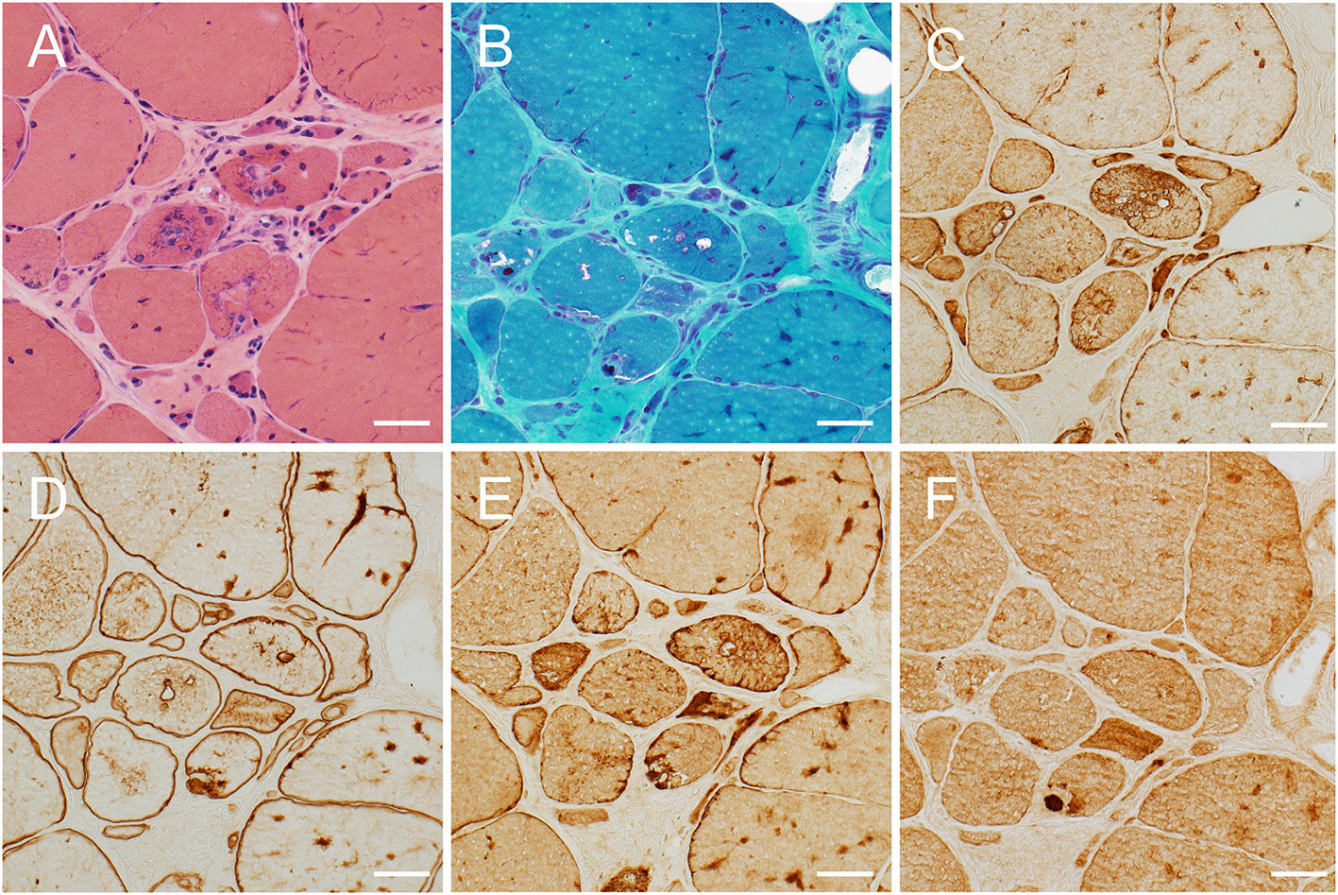
Right quadriceps muscle biopsy findings. Hematoxylin and eosin **(A)** and modified Gomori trichrome **(B)** stained sections demonstrating variation in fiber size, increased endomysial and perimysial connective tissue, rimmed vacuoles, and hyaline inclusions with increased immunoreactivity for desmin **(C)**, dystrophin **(D)**, αB-crystallin **(E)** and myotilin **(F)**, indicating a myofibrillar myopathy. Scale bar = 50 μm.

**Figure 2:**
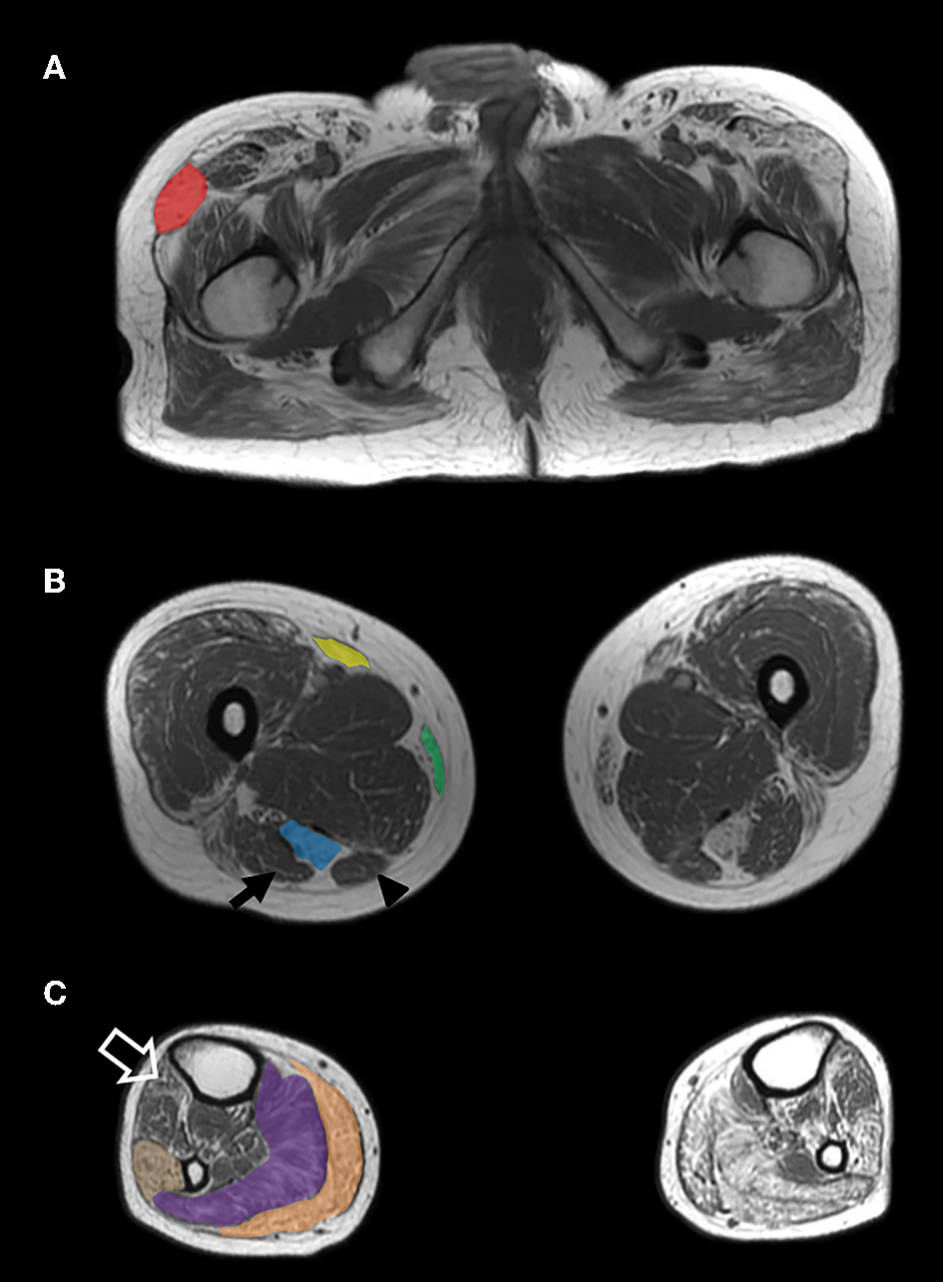
MRI findings. Axiat T1-weighted MRI images of the proximal thigh **(A)**, largest diameter of the mid-thigh **(B)** and largest diameter of the mid-calf **(C)** demonstrate grade IV (>50% of the muscle) atrophy and fatty replacement of the tensor fasciae latae (**A**—red), semitendinosus (**B**—blue), sartorius (**B**—yellow), gracilis (**B**—green), gastrocnemius (**C**—orange), soleus (**C**—purple), and peroneus longus (**C**—tan) muscles. The biceps femoris (**B**—arrow), semimebranosus (**B**—arrowhead), and tibialis anterior (**C**—open arrow) are relatively spared.

Segregation analysis was subsequently performed. The *DES* variant was present in the affected sister and in the brother with elevated CK level, but absent in an unaffected sister. By contrast, the *RYR1* variant did not segregate with the phenotype. A repeat muscle biopsy of the right quadriceps showed findings typical of MFM (Figure 1). A muscle MRI demonstrated atrophy and fatty replacement most severely affecting the tensor fasciae latae, semitendinosus, sartorius, gracilis, gastrocnemius, soleus and peroneus longus muscles (Figure 2). By contrast, the biceps femoris, semimembranosus and tibialis anterior were relatively spared.

## Discussion

Herein, we report a Hmong family with an autosomal dominant distal myopathy and asymptomatic hyperCKemia due to the p.Ser13Tyr missense variant in the desmin gene. The distal-predominant weakness observed in the proband is typical of desminopathy (6). Cardiomyopathy, which is common in desminopathy patients, was absent in this family (6, 7).

The findings observed on muscle biopsy establish the presence of a MFM and thus support the pathogenicity of the mutation in *DES*. The serine residue at position 13 is highly conserved and serves as a phosphorylation site for protein kinase C (8). Its functional importance is demonstrated by the existence of another pathogenic substitution affecting the same amino acid, p.Ser13Phe. Patients carrying this variant typically present with a severe cardiac phenotype, which may occur in isolation or in association with proximal or distal weakness (8–10). The absence of the c.38C>A variant from population databases and its segregation in the family provide additional supportive evidence of its pathogenicity, as do the proband's MRI findings.

Imaging studies have identified two distinct patterns of muscle involvement in MFM. Selective involvement of the semitendinosus, sartorius, gracilis, and peroneus longus muscles is typical of desminopathies and αB-crystallinopathies (11–13). By contrast, a mirror image is seen in myotilinopathies and filaminopathies, in which there is involvement of the biceps femoris, semimembranosus, tibialis anterior, medial gastrocnemius and soleus. Fischer et al. reported that among a group of 46 MFM patients, MRI involvement of the semitendinosus equalling or exceeding the biceps femoris and involvement of the peroneal muscles equalling or exceeding the tibialis anterior predicted the presence of a desminopathy with 100% sensitivity and 95% specificity (12). It should be noted however that selective involvement of the semitendinosus has also been reported in hereditary myopathy with early respiratory failure caused by titin mutations and is therefore not pathognomonic of desminopathy (14). While muscle MRI was successful in distinguishing myotilinopathy from other distal myopathies (5), its sensitivity and specificity for desminopathy in this context remains to be established. In inherited myopathies, MRI should therefore be interpreted in the context of the clinical picture and results of molecular genetic testing. Muscle MRI can also assist in the interpretation of VUS, particularly in genes such as *DES*, in which a distinctive imaging pattern of muscle involvement has been described.

## Data Availability Statement

The datasets generated for this study are available on request to the corresponding author.

## Ethics Statement

Ethical review and approval was not required for the study on human participants in accordance with the local legislation and institutional requirements. Written informed consent for participation was not required for this study in accordance with the national legislation and the institutional requirements.

## Author Contributions

SN: study design, data collection, and drafting of a first version of the manuscript. BH: review of the MRI images and critical revision of the manuscript. EN: study design, data collection, study supervision, and critical revision of the manuscript.

### Conflict of Interest

The authors declare that the research was conducted in the absence of any commercial or financial relationships that could be construed as a potential conflict of interest.
